# Clavicular bone defects managed with free vascularised fibular grafting: evidence to date

**DOI:** 10.1007/s00590-023-03598-8

**Published:** 2023-06-08

**Authors:** Sophia M. Wakefield, Vasileios P. Giannoudis, Peter V. Giannoudis

**Affiliations:** 1 Academic Department of Trauma & Orthopaedic Surgery, School of Medicine, University of Leeds, Clarendon Wing, Floor D, Great George Street, Leeds General Infirmary, Leeds, LS1 3EX UK; 2 NIHR Leeds Biomedical Research Centre, Chapel Allerton Hospital, Leeds, UK

**Keywords:** Clavicle, Non-union, Free fibular vascularised graft, Bone defect, Management

## Abstract

Reconstructive surgery of the clavicle using free vascularised fibula grafting (FVFG) is sometimes required for the management of severe bone loss or non-union. As the procedure is relatively rare, there is no universal agreement on the management and outcome. This systematic review aimed to first, identify the conditions for which FVFG has been applied; second, to gain an understanding of the surgical techniques used; and third, to report outcomes related to bone union, infection eradication, function and complications. A PRISMA strategy was used. Medline, Cochrane Central Register of Controlled Trials, Scopus and EMBASE library databases were interrogated using pre-defined MeSH terms and Boolean operators. Quality of evidence was evaluated based on OCEBM and GRADE systems. Fourteen studies based on 37 patients were identified with a mean follow-up time of 33.3 months. The most common reasons for the procedure were: fracture non-union; tumours requiring resection; post-radiation treatment osteonecrosis and osteomyelitis. The operation approaches were similar, involving graft retrieval, insertion and fixation and vessels chosen for reattachment. The mean clavicular bone defect size was 6.6 cm (± 1.5), prior to FVFG. Bone union occurred in 94.6% with good functional outcomes. Complete infection eradication occurred in those with preceding osteomyelitis. The main complications were broken metalwork, delayed union/non-union and fibular leg paraesthesia (n = 20). The mean re-operation number was 1.6 (range 0–5.0). The study demonstrates that FVFG is well tolerated and has a high success rate. However, patients should be advised about complication development and re-intervention requirement. Interestingly, overall data is sparse with no large cohort groups or randomised trials.

## Introduction

The clavicle is a double curved s-shaped long bone articulating with the sternum medially and acromion laterally, and is stabilised by strong ligaments at either end [1]. Maintaining the integrity of the clavicle is important for normal shoulder function, the avoidance of compression of underlying structures (e.g., brachial plexus and axillary artery) and for optimum respiratory function [2].

Injury and dysfunction of the clavicle most commonly follows a fracture occurring as a consequence of direct trauma to the shoulder [3, 4]. Clavicle fractures are fairly common and account for approximately 2.6–4% of all fractures [5, 6]. In many of these cases, management is non-surgical, but when surgery is required, an open reduction and internal fixation (ORIF) with a plate is most frequently used in the first stages to stabilise the bone and encourage healing [7, 8]. In the UK, surgical intervention for clavicular fractures ranges from 2 to 4% of all fractures [9].

If unsuccessful, the plate is removed and another inserted with an iliac crest autologous bone graft, for enhancement of the fracture healing response. On failing this, reconstructive surgery is then considered. A similar scenario may follow other clavicular disorders, such as tumour infiltration, osteomyelitis or bone necrosis secondary to radiotherapy [10–12], although in these latter cases, reconstructive surgery may be required as an earlier option.

Taylor et al., described a reconstructive approach known as the free vascularised fibula graft (FVFG) to manage significant long bone defects [13]. The principles of this technique relate to the abundant vascularity of the fibula, its similarity in bony shape to the clavicle, and its functional adaptation to the recipient site [14]. However, as the procedure is relatively uncommon [15], there is a paucity of literature specifically describing the outcomes.

The objectives of this review were to: first, identify the conditions for which FVFG has been applied; second, to gain an understanding of the types and choice of surgical techniques used; and third, to report outcomes related to bone union, infection eradication, functional and complications.

## Methods

### Search strategy and criteria

The protocol for this systematic review was based on the preferred reporting items for systematic reviews and meta-analyses (PRISMA) guidelines [16], and was created prior to data extraction. A list of Medical Subject Headings (MeSH) terms and Boolean operators were compiled: (clavicle OR clavicular) AND (non-union OR pseudarthrosis) AND (management OR free vascularised fibular graft). These words were utilised to search Medline (through the PubMed search engine), Cochrane Central Register of Controlled Trials (CENTRAL), Scopus and EMBASE databases. The search strategies implemented are detailed in Appendix 1.

### Study selection

Inclusion criteria were established using the population intervention comparison outcomes (PICO) approach [17]: *Population*: adults (over 18) with clavicular non-union and concomitant osseous defects. There were no limits on sex, ethnicity or co-morbidities of the individuals included. *Intervention*: FVFG. *Comparator*: management strategies used to treat clavicular non-union e.g., ORIF or bone grafting, excluding FVFG. *Outcomes*: the primary outcome measured was bone healing. Secondary outcomes included infection eradication; functional outcomes; and complications including unplanned re-operations. Exclusion criteria included: reviews, editorials and viewpoints, subjects aged 16 years or less, congenital cases of clavicular non-union and cases in which a complete neo-clavicle was required.

All studies were considered for eligibility, with no restrictions on publication date or language applied. Titles and abstracts were screened for relevance prior to full inspection. The reference lists of all eligible studies were reviewed to isolate any articles that may have been missed in the initial database search. Duplicate articles between the databases were removed and the full texts of all studies meeting the inclusion criteria were obtained. To increase the reliability of data extraction, two reviewers blindly performed the study selection and data extraction. Any disagreements between reviewers were resolved with discussion with the senior author.

### Data collection

Data was extracted and collated using a purpose-designed Microsoft Excel spreadsheet. The following data were recorded: (1) study characteristics (study design, sample size); (2) patient demographics and baseline characteristics (age, sex, co-morbidities); (3) initial clavicular injury prior to FVFG intervention (cause of bone defect, presence of infection, number of surgical procedures, type of procedures, size of bone defect); (4) surgical procedure utilised to harvest and transfer FVFG (operation techniques for graft insertion and vascular anastomosis); (5) outcome measures (bone union, infection eradication, functional outcomes, complications).

### Assessment of methodological quality

Methodological quality of the included studies was assessed and graded using the OCEBM ‘Levels of Evidence’ guidelines [18].

The overall quality of evidence in this systematic review was evaluated using the grading of recommendations, assessment, development and evaluation (GRADE) system [19]. Recommendations were classified as either high, moderate, low, or very low according to the authors’ interpretation of the true effect in the study compared to the estimated effect. This approach involved grading the evidence included based on the criteria for risk of bias, imprecision, inconsistency, indirectness and publication bias.

### Statistical analysis

Descriptive statistical analysis (e.g., mean ± standard deviation (SD), mean ranges, ratios and percentages) was collated and reported in this study.

## Results

### Search results

**Fig. 1:**
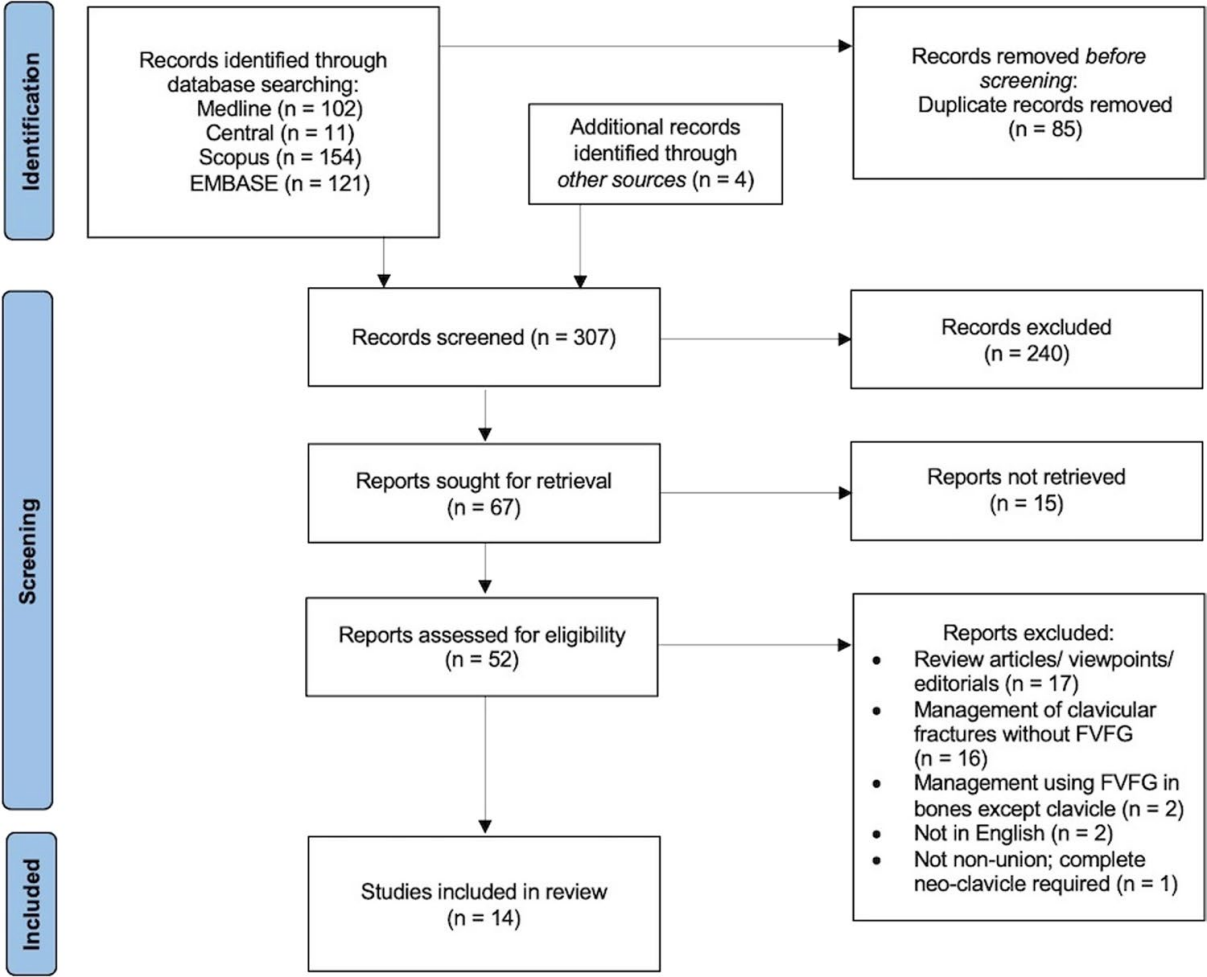
Preferred reporting items for systematic reviews and meta-analyses (PRISMA) flowchart

The PRISMA flowchart is shown in Fig. 1.

102 Medline articles, 11 CENTRAL articles, 154 Scopus articles and 121 EMBASE articles were obtained. In addition, a further search of records yielded an additional 4 studies; this provided a baseline of 392 studies in total. Upon removal of duplicates between the databases, the overall articles screened were 307. These were then narrowed to 67 upon title and abstract screening for relevance, with 52 articles assessed for eligibility into this study. The full texts of all 14 studies meeting the inclusion criteria were obtained [20–33].

Table 1 provides an overview of the studies included in the systematic review. Of the 14 studies included in the analysis, 5 were case series [21–23, 25, 30], 4 were case reports [26–28, 33], 4 were cases within research articles [20, 29, 31, 33], and 1 was a technical note [24]. Overall, 37 individuals treated with FVFG were included in our study. The mean participant age in the studies was 44.8 (± 12.8) years old (mean range 17–68), and the mean follow-up time was 33.3 (± 28.4) months (mean range 3 months–10 years) post-operatively.

**Table 1:** Summary of study, patient, and initial injury characteristics

Author	Study characteristics		Patient characteristics			Initial clavicular injury (prior to FVFG)				
	Design	No. of patients	Mean age ± SD (range)	Ratio M:F	Co-morbidities	Cause(s) of bone defect	Infection present? Y/N	Mean no. of surgical procedures ± SD (range)	Type(s) of surgical procedure	Mean bone defect size ± SD (cm) (range)
Wood. [20]	Cases within research article	2	Unreported	Unreported	Unreported	2 fracture NU	N	Unreported	Unreported	6.0
Momberger et al. [21]	Case series	3	32.0 ± 13.0 (19.0–45.0)	01:02	Unreported	3 persistent fracture NU (3 falls)	N	3.7 ± 1.2 (3.0–5.0)	3 ORIF + plate + ICG, 1 compression screw + grafting, 2 partial resection, 1 IM pin + segmental allograft, 1 further bone grafting	5.3 ± 2.5 (3.0–8.0)
Erdmann et al. [22]	Case series	2	46.5 ± 0.7 (46.0–47.0)	01:01	2 smokers, 1 alcohol-consumer, 1 HTN, 1 depression, 1 hepatitis	1 persistent fracture NU (RTA), 1 OM following fracture NU (RTA)	Y (MRSA)	4.5 ± 3.5 (2.0–7.0)	1 ORIF + 1/3 semi-tubular plate, 1 ORIF + low-compression titanium plate + synthetic bone grafting, 1 ORIF + ICG + partial pectoralis major muscle transfer, 1 ORIF + proximal tibial cancellous bone grafting, 1 ORIF + ICG	4.5 ± 0.7 (4.0–5.0)
Krishnan et al. [23]	Case series	8	52.5 ± 4.7 (46.0–59.0)	06:02	Unreported	8 pseudoarthrotic NU + symptomatic brachial plexus compression	N	> 2.0 (exact number unreported)	Wiring + plating, plating + graft + plate fixation, all 3 surgical procedures combined	Unreported
Lenoir et al. [24]	Technical note	2	50.5 ± 3.5 (48.0–53.0)	01:01	2 smokers	1 fracture NU, 1 fracture NU, with local infection (Hx: radiotherapy for Hodgkin's lymphoma)	Y (Staph aureus)	6.0	2 ORIF, 2 ICG + locking plate, 1 osteosynthesis with local implantation (BMP-7)	6.0 ± 0.7 (5.5–6.5)
Abarca et al. [25]	Case series	4	55.3 ± 8.7 (45.0–65.0)	01:03	1 smoker	1 tumour (right apex lung carcinoma), 2 persistent fracture NU, 1 OM after fracture NU	Y	3.0 ± 1.4 (1.0–4.0)	1 carcinoma resection, 2 ORIF, 3 ICG + compression plate,	7.8 ± 3.1(5.0–12.0)
Ye et al. [26]	Case report	1	52	01:00	1 smoker	1 tumour (5 cm recurrent dermatofibrosarcoma)	N	1	Local excision	9.5
Choke et al. [27]	Case report	1	39	0:1	Unreported	1 OM	Y (TB)	Unreported	“Multiple debridements"	8
Arenas-Miquelez et al. [28]	Case report	1	50.0	0:1	Unreported	1 persistent fracture-dislocation NU	N	Unreported	“Multiple revision surgeries”	5.3
Goormans et al.[29]	Case within research article	1	17.0	0:1	Unreported	1 OM following fracture NU (fall)	Y (Pseudomonas sp)	3.0	1 screw osteosynthesis, 1 revision plate osteosynthesis, 1 debridement + irrigation	8
Petje et al. [30]	Case series	7	40.0 ± 16.0 (19.0–65.0)	05:02	1 smoker	6 fracture NU, 1 OM	Y	1.9 ± 0.7 (0–3.0)	7 ORIF + plating	6.3 ± 1.0 (5.0–7.8)
Lim et al. [31]	Cases within research article	3	46.3 ± 5.0 (41.0–51.0)	02:01	1 smoker	1 ORN (nasopharyngeal carcinoma), 2 fracture NU	N	Unreported	Unreported	6.3 ± 2.1 (4.3–8.5)
Claxton et al. [32]	Case within research article	1	33.0	01:00	Unreported	1 tumour (plasmacytoma)	N	1.0	1 tumour resection	Unreported
Wu et al. [33]	Case report	1	68	0:1	Unreported	1 ORN (breast cancer)	N	2	1 mastectomy	Unreported

*SD* standard deviation, *M:F* males: females, *FVFG* free vascularised fibular graft, *HTN* hypertension, *NU* non-union; *OM* osteomyelitis, *ORN* osteoradionecrosis, *RTA * road traffic accident, *Hx* history, *MRSA* methicillin-resistant *Staphylococcus aureus*, *ORIF* open reduction and internal fixation, *ICG* iliac crest graft, *IM* intramedullary, *BMP-7* bone morphogenetic protein-7

### Methodological quality

OCEBM ‘Levels of Evidence’ (Appendix 2) demonstrated the overall level of evidence of all 14 studies [20–33], included as “Level IV”. This is due to all research being case series [21–23, 25, 30], case reports [26–28, 33], case reports within research articles [20, 29, 31, 33], and a technical note [24].

In addition, Appendix 2 provides an overview of the GRADE analysis assessment, which demonstrated the quality of evidence to be Low for all analyses, as there were no large observational studies or randomised controlled trials on this subject.

### Patient demographics and baseline characteristics

Of the 37 individuals reported in this review with a mean patient age of 44.8 (± 12.8) years old, 19 were men and 16 women; the sex of 2 cases was unreported (1.2:1 male to female ratio) (Table 1).

With regards to co-morbidities, there were 8 tobacco smokers [22, 24–26, 30, 31], 1 patient with excess alcohol consumption [22], 1 hypertensive patient [22], 1 individual living with depression [22] and another with hepatitis [22].

### Initial clavicular injury and surgical procedures prior to FVFG intervention

The causes of clavicular bone defect have been classified into four main categories: fracture non-union, tumours, osteoradionecrosis following radiation therapy for tumour treatment and osteomyelitis.

In this review, there were 27 persistent non-unions secondary to fracture (e.g., from fall onto ipsilateral shoulder, road traffic accident, gunshot wound) [20–25, 28–31]; 3 tumours requiring resection (1 lung apex carcinoma, 1 recurrent dermatofibrosarcoma, 1 plasmacytoma) [25, 26, 32]; 2 cases of osteoradionecrosis (following radiotherapy of: 1 nasopharyngeal carcinoma, 1 breast cancer) [31, 33]; and six cases of clavicular osteomyelitis (1 tuberculosis, 1 Methicillin-resistant *Staphylococcus aureus* (MRSA), 1 *Staphylococcus aureus*, 1 Pseudomonas species, 2 further unidentified species) [22, 24, 25, 27, 29, 30], (Table 1).

Prior to surgical management using FVFG, the mean number of previous operations was 2.5 (± 1.8), with a mean range of 0.5–6 procedures, based on the information provided (Table 1). Management strategies of these cases prior to the fixation of the bone defect with fibular grafting varied, and included both conservative and surgical fixation. First-line conservative management of these clavicular injuries were reported in 8/37 cases [21, 22, 24, 26], through the use of a figure-of-8 splint. In contrast, ORIF was the most common surgical procedure, accounting for 70.3% (26/37) of all operative techniques attempted before FVFG [20–26, 28–33]. ORIF was performed using: a compression plate alone (n = 12) [22, 24, 25, 30], plate with iliac crest graft (ICG) (n = 9) [21, 22, 24, 25], plate with synthetic graft (n = 1) [22], plate with tibial cancellous graft (1) [22], or plate with ICG and the addition of a pectoralis muscle flap (n = 1) [22]. In addition, ORIF was also achieved with a wire, plate and graft (n = 1) [23] or a wire and plate alone (n = 1) [23], screw fixation (n = 2) [21, 29] and the implementation of bone morphogenetic protein-7 (BMP-7) (n = 1) [24]. Further techniques that were implemented were resection/tumour excision (n = 6) [21, 25, 26, 32, 33], debridement (n = 1) [27], intramedullary (IM) pin insertion with segmental allograft (n = 1) [21], and further grafting (n = 1) [21]. In five cases, precise detail describing the technique of primary fixation performed was not provided [27, 28, 31] (Table 1).

The mean clavicular bone defect size was 6.6 cm (± 1.5), prior to vascularised fibular grafting. The mean size of these defects in the sample ranged from 4.5 cm to 9.5 cm (Table 1).

### Surgical procedure for FVFG harvesting and transfer

The reported surgical procedures followed similar principles but with some variations, (Table 2). All but one case reported the grafts being stabilised using internal fixation with plate and screws [20–28, 30–33]. The arterial and venous anastomoses are documented in Table 2.

**Table 2:** Characteristics of surgical procedure and outcome measures

Author	Surgical procedure for FVFG		Outcome measures					Mean F/U ± SD (months) (range)
	Operative technique for graft implantation	Vessel anastomosis (artery + vein)	Bony union achieved (%)	Infection eradication? Y/N	Functional outcomes	Complications	No. of re-operations	
Wood. [20]	1 ORIF + screws, 1 ORIF + 2 plates	Unreported	50	–	1 symptomatic flail clavicle	Pain, NU	Unreported	30.0 ± 19.8 (16.0–44.0)
Momberger et al. [21]	2 ORIF + dynamic compression plate, 1 had 4 cortices fixed in proximal + distal native clavicular segments + graft1 6.5 mm lag screw attaching FVFG to coracoid process	1 thyrocervical artery + EJV	100	–	2 pain-free, full shoulder ROM, 1 almost at full ROM	1 non-displaced stress fracture, 1 broken coracoid lag screw, 1 pain in neo-ACJ, 1 ulnar-distribution dysesthesia	4	22.6 ± 2.8 (20.0–24.0)
Erdmann et al. [22]	1 ORIF + dynamic compression plate + titanium screws laterally and medially, 1 "telescopic" style approach (lateral + medial clavicle bone ends burred to inset the FVFG into recipient lateral clavicle defect) + 2.5 mm dynamic compression plate	1 TCA + TCV, 1 Thyrocervical trunk + IJV + superficial cutaneous branch of external jugular system	100	Y	2 full shoulder ROM, no signs of infection	1 complicated post-operative respiratory course	0	3
Krishnan et al. [23]	8 ORIF + plate + screw	8 branch of thoraco-acromial trunk (arterial + venous supply)	100	–	Improvement in sensory (7/8), motor (8/8), pain (8/8) modalities. Improvements also observed in F-Wave (3/6), EMG (4/4), NCV (5/8)	3 bleeding revisions, 1 fistula formation, 2 removal of osteosynthetic materials	5	38.5 ± 16.1 (18.0–60.0)
Lenoir et al. [24]	2 ORIF + plate	2 Superficial TCA + branch of external jugular system	100	Y	2 pain-free, full shoulder ROM, Constant Shoulder Score improved for both patients: 48 to 96 and 45 to 90	1 soft tissue irritation (plate), 1 delayed union at junction between the medial clavicle + FVFG	1	16.0 ± 5.7 (12.0–20.0)
Abarca et al. [25]	1 proximal cleidosternal screw + distal dynamic compression plate, 1 IM pinning + metallic band tension on both clavicular ends (Brunelli method), 1 ORIF + rigid plate + screws, 1 ACJ stabilisation + ORIF (plate, screws + K-wire)	1 thyrocervical trunk + adjacent vein, 1 thoracoacromial artery + cephalic vein, 2 TCA + TCV	100	Y	3 pain-free, good-normal shoulder ROM (can perform ADLs), 1 extensive physiotherapy	1 local infection + poor flap vascularisation, 1 scar dehiscence (gentamicin required) 1 SC pain, 1 extensive bone defect	3	21.8 ± 19.2 (6.0–48.0)
Ye et al. [26]	ORIF + straightened Richardson dislocation hook plate + unicortical screws + split short head of biceps tendon (to reconstruct CC ligament)	1 TCA + EJV	100	–	Pain-free symmetrical full shoulder ROM, Constant Shoulder Score = 77	Diminished abduction strength (deltoid muscle removal)	0	120
Choke et al. [27]	1 ORIF + 9-hole locking plate,at medial end, “new” joint was created using 1 mm steel wire + Ethibond 1/0 suture	1 DSA + EJV + IJV	100	Y	1 pain-free, full shoulder ROM (140 degrees shoulder abduction, 110 degrees of shoulder flexion + 70 degrees of shoulder extension), Likert Scale = 14/20, QuickDash assessment = 8.62 (minimal disability)	–	0	18
Arenas-Miquelez et al. [28]	1 ORIF + customized length 3.5 mm locking compression plate hook plate,ACJ stabilisation with BiPOD (biplanar) technique	1 branch of thoraco-acromial trunk (arterial + venous supply)	0	–	1 initial pain reduction + improved neuro Sx. Recurrence of intermediate pain + neuro Sx over the clavicle	1 persistent NU, 1 persistent Sx (e.g., pain)	> 2(Confirmed figure unreported)	48
Goormans et al. [29]	1 3-stage technique:debridement + irrigation, resection, FVFG implantation	1 thoracoacromial artery branch + cephalic vein (1)	100	Y	1 pain-free, full shoulder ROM + full return to activities	–	0	24
Petje et al. [30]	7 dynamic compression plates (5 LC-DCP Synthes 8-hole plate, 2 Königsee Locking 10-hole plate) + graft + monocortical screws	7 superficial branch of TCA + 2 adequate veins	100	Y	7 Tang classification: 4 excellent, 2 good, 1 fair	7 mild paraesthesia (donor leg), 7 weakness of long toe flexors and extensors, discomfort on walking, broken plates	4	31.0 (22.0–54.0)
Lim et al. [31]	1 ORIF + reconstruction plate, 2 unreported	3 thoracoacromial/ superior thyroid/ TCAs + thoracoacromial and EJVs	100	Y	3 1 MRC score improvement from: 2/5 to 3/5 (1), 1/5 to 4/5 (1), 2/5 to 5/5 (1)	1 skin infection + partial donor site skin graft loss, 1 venous kinking	Unreported	Unreported
Claxton et al. [32]	1 ORIF + dual locking plating + screws	Unreported	100	–	MSTS score improvement from: 37 to 70%, improved use of arm	Painful hardware loosening	1	36
Wu et al. [33]	1 ORIF + plate at distal end + wire at proximal end	1 thoracoacromial artery branch + cephalic vein	100	–	1 pain-free, full shoulder ROM, complete wound healing,equal grip strength bilaterally	–	0	24

*FVFG* free vascularised fibular graft, *F/U* follow-up, *SD* standard deviation, *ORIF* open reduction and internal fixation, *EJV* external jugular vein, *IJV* internal jugular vein, *ROM* range of movement, *ACJ* acromioclavicular joint, *Sx* symptoms, *NU* non-union, *TCA* transverse cervical artery, *TCV* transverse cervical vein, *CC* coracoclavicular, *ADL* activities of daily living, *SC* sternoclavicular, *EMG* electromyography, *NCV* nerve conduction studies

### Outcome measures

The primary outcome measure of successful bone union occurred in 35/37 (94.6%) individuals [20–27, 29–33]. When infection was a cause of non-union, the results demonstrated eradication in 100% of cases (6/6) [22, 24, 25, 27, 29, 30]. Table 3 demonstrates the range of scoring systems used to measure functional outcomes. The results of these functional scoring assessments are found in Table 2.

**Table 3:** Scoring systems to assess functional outcomes

Scoring system for outcome measure	Number of studies
Shoulder range of motion (ROM)	9
Sensory/ motor/pain deficits or improvements	8
Constant-Murley Shoulder Outcome Score	3
Effects on activities of daily living (ADLs)	3
Visual Analogue Scale (VAS) score	1
QuickDash assessment for disability	1
Likert scale for flap appearance	1
Shoulder Motor Function (MRC)	1
Tang score	1
Musculoskeletal Tumor Society (MSTS) scoring	1
Nerve conduction studies	1

### Complications and follow-up

There were a variety of post-operative complications noted in the reports included in this study (Table 2). 10 patients experienced pain (8 fibula, 2 clavicle) [21, 25, 28, 32], 8 paraesthesia (7 fibula, 1 ulnar nerve distribution) [21, 30] and 7 patients displayed weakness, in particular the long toe flexors and extensors [30]. Furthermore, 2 patients demonstrated persistent clavicular non-union [20, 28], 2 had delayed union (but eventually united) [24, 25] and 1 re-fracture was observed [21]. Additional complications included: 2 skin infections managed with antibiotics, 1 fistula formation, 1 scar dehiscence and 1 venous kinking [23, 25, 31]. The mean number of re-operations was 1.6 (± 2.0), with a range of 0–5.0 additional procedures. These further operations were due to broken metalwork, pain, infection, bleeding and cosmesis, and involved: plate removals (n = 7) [21, 28, 30, 32], screw removals (n = 3) [21, 25, 32], further cancellous grafting (n = 4) [24, 30], bleeding revisions (n = 3) [23] and the removal of an infected flap and debridement (n = 1) [25] (Table 2). The mean follow-up time was 33.3 months (± 28.4) (range 3 months–10 years).

## Discussion

The treatment of large bone defects remains a challenge for reconstructive surgeons. Usually, this group of patients has been through a prolonged clinical journey having undergone several operations that have failed to address the original problem, usually being fracture non-union and/or chronic osteomyelitis. The need to resect the avascular, dead bone leads to bone loss and the development of bone defects.

Evidence was sought for the use of a vascularised fibular bone graft for the management of significant clavicular bone defects requiring reconstructive surgery. We examined the aetiology, patient characteristics, the variations in surgical technique and outcomes.

Overall, there was a slightly higher predisposition towards males requiring this surgery. The mean age was 44.8 years which may reflect a higher rate of falls, an increasing risk of bone pathologies or less preponderance to healing when compared to a younger age group [4]. Direct trauma accounted for approximately 80% (30/37) of the cases as the primary mechanism of injury. In these cases, fibular graft surgery often followed several preceding operative interventions (mean previous operations 2.5), such as an ORIF with plating, bone grafting (often from the iliac crest) and debridement. The remaining cases mostly related to infiltrative and/or destructive disease processes, such as that caused by tumour, radiotherapy or infection; in these situations, the procedure was more likely to be done as an earlier intervention [34, 35].

The mean cortical defect size across studies was 6.6 cm (range 4.5–9.5 cm); their relatively large size reflecting the need for a more aggressive approach. In some cases, the large size of the defect reflected the consequences of multiple previous operative resections and debridements of the bone ends from previous attempts at surgery or because the lesion was primarily large e.g. due to neoplasm.

Although the general principles of harvesting and implanting a graft was similar between studies, there were variations in the operation technique. The choice of a specific technique might be dependent on the degree of bone loss, underlying reasons for the bone loss as well as the personal preferences of the surgeon.

The reported overall outcome of a fibular graft was 94.6% (35/37) for a successful union. Eleven different functional outcome measures were employed across the studies highlighting a lack of standardisation. The reported complications related to either the clavicle itself (21 patients) or the fibular donor site (15 patients). With respect to the clavicle, most complications related to the metalwork and vascular tree. In contrast, paraesthesia and weakness were the main consequences of the fibular procedure [30]. The mean number of re-operations was 1.6, with a range of 0 to 5.0 additional procedures. It is therefore pertinent for surgeons to warn patients of an increased risk of re-interventions and chronic pain/ paraesthesia to the donor or recipient site.

The main limitation of the study is the relatively small sample size which has prevented formal statistical analysis. It is also unknown how common this procedure is performed within practice and whether only cases with a positive surgical outcome are published. It was also noted that there were differences in what data was reported within studies resulting in variability in some demographic and outcome data. The two cases that failed to unite did not provide data on co-morbidities or previous surgical procedures [20, 28].

The strengths of the paper relate to it being a systematic review which followed a structured strategy for data collection and analysis. Integrity of the data was optimised by discussions of the articles between authors. During preparation of this manuscript, another systematic review on the same topic was published [33]. This was based on data up to January 2020 from only 3 search engines (including Google scholar) and included contrary to our strategy, data on paediatric and congenital cases [33]. Despite this, we feel that our study provides further information to the subject area for reconstruction of clavicular defects in adult patients with similar aetiopathogeneses related to fracture non-union and chronic osteomyelitis.

In summary, this study has highlighted that the use of a FVFG, when applied in specific situations, often has successful clinical, functional and radiological outcomes. However, the lack of standardisation of procedures and outcome measurement, and the available small number of patients reported makes it challenging to provide a comprehensive evaluation of the technique. Further studies with larger patient sample sizes are desirable to provide more robust evidence and facilitate a meta-analysis in this field.

## Appendix 1: Tables showing the Medline, CENTRAL, Scopus and EMBASE database search protocols used

**Table:**

Search	Number of results
*Medline*	
(1) “clavicle” OR “clavicular”	12,461
(2) “non-union” OR “pseudarthrosis”	13,035
(3) “management” OR “free vascularised fibular graft”	3,656,271
(4) 1, 2 AND 3	102
*CENTRAL*	
(1) “clavicle” OR “clavicular”	849
(2) “non-union” OR “pseudarthrosis”	617
(3) “management” OR “free vascularised fibular graft”	163,683
(4) 1, 2 AND 3	11
*Scopus*	
(1) “clavicle” OR “clavicular”	28,044
(2) “non-union” OR “pseudarthrosis”	40,675
(3) “management” OR “free vascularised fibular graft”	6767
(4) 1, 2 AND 3	154
*EMBASE*	
(1) “clavicle” OR “clavicular”	16,224
(2) “non-union” OR “pseudarthrosis”	19,650
(3) “management” OR “free vascularised fibular graft”	2,961,975
(4) 1, 2 AND 3	121

## Appendix 2: OCEBM and GRADE methodological systems

All primary research articles obtained for this systematic review were case series, case reports, case reports within research studies or a technical note. There are no current large cohort studies or randomised controlled trials (RCTs) describing the surgical technique of free vascularised fibular grafting for clavicular bone defects.

**Table:**

Author	Design of study	OCEBM level of evidence [18]	GRADE assessment [19]
Wood [20]	Cases within research article	Level IV	Low
Momberger et al. [21]	Case series	Level IV	Low
Erdmann et al. [22]	Case series	Level IV	Low
Krishnan et al. [23]	Case series	Level IV	Low
Lenoir et al. [24]	Technical note	Level IV	Low
Abarca et al. [25]	Case series	Level IV	Low
Ye et al. [26]	Case report	Level IV	Low
Choke et al. [27]	Case report	Level IV	Low
Arenas-Miquelez et al. [28]	Case report	Level IV	Low
Goormans et al. [29]	Case within research article	Level IV	Low
Petje et al. [30]	Case series	Level IV	Low
Lim et al. [31]	Cases within research article	Level IV	Low
Claxton et al. [32]	Case within research article	Level IV	Low
Wu et al. [33]	Case report	Level IV	Low
